# Abiotic Stress Tolerance Boosted by Genetic Diversity in Plants

**DOI:** 10.3390/ijms25105367

**Published:** 2024-05-14

**Authors:** Andrés J. Cortés

**Affiliations:** 1 Corporación Colombiana de Investigación Agropecuaria AGROSAVIA, C.I. La Selva, Km 7 vía Rionegro—Las Palmas, Rionegro 054048, Colombia; acortes@agrosavia.co; 2 Facultad de Ciencias Agrarias—de Ciencias Forestales, Universidad Nacional de Colombia—Sede Medellín, Medellín 050034, Colombia; 3 Department of Plant Breeding, Swedish University of Agricultural Sciences, Lomma 23436, Sweden

Plant breeding [1], conservation [2], and restoration [3] efforts necessitate the development of novel adaptive sources to cope with increasing abiotic pressures [4]. However, standing genetic diversity for tolerance to abiotic stress is often lacking in founder genepools [5]. Therefore, this Special Issue aims to compile innovative research on the exploration, leveraging, and utilization of plant genetic diversity to improve abiotic stress tolerance traits using a diverse array of techniques and perspectives. In this exquisite compilation, the contributing authors have merged modern methodological achievements in molecular biology, genomics, bioinformatics, and biotechnology to reconstruct the genomic bases and ecological drivers of tolerance to key abiotic stresses such as drought, heat, salinity, and soil toxicity. The research teams successfully pinpointed how cryptic pockets of genetic diversity provide hidden adaptations, genotypes, and alleles that allow organisms to efficiently withstand abiotic pressures. Ultimately, the works gathered in this collection propose innovative achievements that will lead to new interdisciplinary avenues where plant breeding, restoration ecology and conservation genetics interplay.

## 1. Polygenic Diversity for Abiotic Stress Tolerance

Tolerances to abiotic stresses have typically been regarded as complex quantitative traits conferring adaptation [6]. In other words, in contrast with biotic resistances [7,8], abiotic responses recruit more infinitesimal effects across hundreds and thousands of variants [9]. This type of polygenic model has two broad implications when it comes to addressing the adaptive potential of plant species, and its inheritance [10,11]. First, it leads to substantial G × E effects [12], some of them modulated by environmental reaction norms [13], while others could be the result of alleles exhibiting antagonistic pleiotropy [14] or, alternatively, conditional neutrality across environments [15]. Second, having abundant variants with subtle additive genetic effects conveys greater potential in terms of the evolvability of the system [16]. After all, natural selection encounters a rich playground with infinite genic re-combinations [17].

However, when overall natural genetic variation is depleted by demographic and selective forces [18], population genetics theory predicts that genetic drift will lead to bottlenecks in low-frequency quasi-neutral variants [19], which by definition are those underlying the polygenic model [20]. In practice, Fisherian polygenic effects follow a decaying exponential distribution [21], with many low-effect variants, and very few medium- and high-effect alleles. Currently, depletion in the adaptive potential is driven by stronger effects from the causal agents, including (i) demographic instability due to the effects of climate change, and (ii) intrinsic strong positive selection for very few traits in the founding populations of modern crops [22], relict and secluded natural populations [23], and the seedbank of restoration areas [3].

Jeopardized genetic variation will therefore not take long to undermine the foundation of the adaptive genetic response itself [24]. Fortunately, throughout this collection, the authors have offered insights to bridge this gap regarding the lack of genetic diversity for abiotic stress tolerance (Table 1). They have amassed a rich repertoire of bottom-up examples regarding in silico mining of standing genetic variation for stress responses [25]. The baseline mapping, made possible by the explosion of genomic resources for non-model and orphan organisms [26], is followed up by the reconstruction [27] and prediction [28] of its genomic architecture using last-generation linkage disequilibrium mapping [29,30]. Finally, downstream greenhouse-based validation of the rescued variants for adaptation to abiotic stresses is carried out with expression analysis and transgenesis.

The widespread availability of plant genomes [39] enables the systematic characterization of predicted genic features in terms of their functional and phenotypic correlates. Within this exciting framework, Huang et al. [31] achieved in silico prediction of the role of NAC transcription factors (TFs) to face a wide spectrum of abiotic stresses [40], including drought and salinity, among others [41], in *Apocynum venetum* (Apocynaceae), an important source of natural fiber, ideally as restoration species for saline–alkaline soils. Specifically, the authors offered a comprehensive analysis of the TFs involved in the abiotic stress response in these species. The study identified and clustered 74 AvNAC proteins into 16 subgroups. Notably, genomic features as segmental duplication events [42] and strong purifying selection [43] were the main forces responsible for the growth of the AvNAC TF family. In turn, the regulatory complexity of AvNAC promoters was found to be driven by cis-elements [44]. Gene expression and protein interaction predictions support the importance of AvNAC58 and AvNAC69, which are important regulators of the responses to salt stress and drought, with implications for the trehalose metabolism pathway [45]. The work not only expands our knowledge of the stress-response mechanisms in *A. venetum*, but also reinforces how in silico approaches can deliver valuable insights to optimize crop resilience in response to climate change [46,47].

When the precise location of the candidate genes is furtive, genetic mapping offers a straightforward and utile pipeline [48]. With this in mind, Ravelombola et al. [32] and Wirojsirasak et al. [33], respectively, mapped salt and drought tolerance in cowpea (*Vigna unguiculata*) and sugarcane (*Saccharum officinarum*), utilizing whole-genome and target enrichment sequencing. In the first study, Ravelombola et al. [32] focused on the molecular pathways with the aim of controlling cowpeas’ response to salt stress during the seedling stage. Target SNP markers and candidate genes associated with the leaf chlorophyll content and damage scores phenotypes were identified on chromosomes 1 and 2, and included genes for potassium channels and GATA TFs, respectively. Meanwhile, Wirojsirasak et al. [33] inspected 18 traits and 649 candidate genes and captured 197 significant marker-trait associations (MTAs) during the drought response of sugarcane. Interestingly, these MTAs exhibited unique genetic variations associated with physiological adaptation, phytohormone metabolism, and drought response mechanisms. They were also found consistently in both water-stressed (WS) and non-stressed (NS) environments. The discovery of non-antagonistic pleiotropy for various environmental conditions opens up a promising pathway for stability in trait selection, providing a comprehensive strategy to improve the adaptability of sugarcane cultivars to a wide range of environmental conditions without inflating the G × E [49]. Both research works reinforce polygenic bases for abiotic stress responses, salinity and drought included, which convey technical challenges to ensure rapid and consistent genetic gains via molecular breeding for cowpea and sugarcane sustainable farming in the face of weather uncertainty [50].

Mapping efforts such as those described above may be prone to spurious MTAs due to demographic stratification [51] segregating concurrently with the target phenotypes [52], not to mention the intrinsic multiple testing inherent to the mapping algorithms, and proportional to the number of markers being surveyed [53,54]. In light of this, rigorous validation of candidate associations is a non-negotiable requirement [55] before one can proceed with more downstream applications. It is as part of this corroboration endeavor that Zhao et al. [34], Ma et al. [35], and Guo et al. [36] envisioned their works, using alfalfa (*Medicago sativa*), *Phoebe bournei* (a widely conserved tree in China because of its economic and ecological value), and sorghum (*Sorghum bicolor*) as study organisms for multiple abiotic stresses, such as salt (all three species), drought (the former two taxa), and heat (the second species). Performing integrated assessment of multiple stresses such as these also enables long-standing questions concerning the scale of molecular pleiotropism beneath concomitant abiotic stresses to be addressed [56], a matter which has already been approached in this Special Issue by Wirojsirasak et al. [33] in a multi-environment framework [12], but has yet to be addressed in a multi-trait setup [57].

## 2. Pleiotropism Underlying Concerted Responses to Abiotic Stresses

Zhao et al. [34] carried out the first attempt to concurrently study salt, drought, and ABA stresses in alfalfa by functionally validating 15 *MsMsr* (methionine sulfoxide reductase) genes with qRT-PCR. The investigation of the alfalfa *Msr* gene family represents a major advancement in our knowledge of the plant’s abiotic stress response via pleiotropism [56]. These *MsMsr* genes clearly show dynamic expression patterns in different tissues under abiotic stress conditions, as demonstrated by transcriptional analysis. This thorough characterization emphasizes how essential *MsMsr* genes are to alfalfa’s ability to withstand environmental stressors in a combined manner. This work opens the door to more focused indirect marker-based breeding techniques [58,59], which will increase the yield and quality of this key crop. Similarly, Ma et al. [35] considered 38 putative LBD (lateral organ boundary domain) gene sequences (5 of which were targeted with qRT-PCR) in *P. bournei*, a target for conservation, in a multi-stress frame. Meanwhile, in another work, the molecular pleiotropism was reinforced across multiple abiotic stresses [60]. Specifically, PbLBDs were found to be pleiotropic for cold, heat, drought, and salt stress response. This study is noteworthy because it reveals the many ways in which LBD TFs react to different types of light and abiotic stressors, indicating the complex functions these TFs play in plant adaptation and survival. The work also establishes a feasible roadmap for further research on the functional significance of LBD genes, potentially informing strategies for its mining, unlocking, and utilization in other species [61].

In terms of candidate gene functional validation [62], Guo et al. [36] also corroborated our knowledge on the genomic architecture for key abiotic stresses by performing transcriptome profiling and qRT-PCR for salt tolerance in sorghum. Ultimately, research investigating how sorghum reacts to salt stress sheds essential light on the plant’s extraordinary capacity to adapt to harsh conditions [63]. The team further positioned the cultivar “Lvjuren” as a model to comprehend how ion homeostasis can be maintained in saline environments. The results suggest that sorghum can withstand and even reduce the effects of ion toxicity by effectively excluding sodium from shoots and selectively accumulating chloride in leaf sheaths, while maintaining nitrate homeostasis in leaf blades [64]. By highlighting important genes and TFs essential to ion transport and regulatory processes, transcriptome profiling can provide us with information on the molecular mechanisms behind salt tolerance, an experimental pipeline that is also applicable in other species. This methodologically innovative work not only broadens our comprehension of plant resilience but also provides a strong foundation for improving agricultural sustainability in salt-prone areas.

Last but not least, Wang et al. [37] also studied toxicity in sorghum, this time for cadmium (Cd), yet went a step further in terms of the candidate gene validation path by recurring to trans-genesis for functional corroboration. Specifically, the team overexpressed *SbEXPA11* to validate its functionality during Cd exposure as a target for phytoremediation. This potential application is highly feasible, at least as proof of concept, because through examining the expansin gene family’s reaction to heavy metal exposure, Wang et al. [37] corroborated that *SbEXPA11* possesses the capacity to accumulate and detoxify Cd. These researchers further clarified how *SbEXPA11* conveys Cd tolerance by scavenging reactive oxygen species (ROS), and increasing the production of antioxidant enzymes. The transgenic sorghum line exhibited remarkable improvements in photosynthetic efficiency, which resulted in increased biomass production and an effective decrease in soil Cd. Therefore, this exciting research provides an insight into how pleiotropic responses in abiotic stress tolerance may eventually allow us to bio-engineer phytoremediary variants [65] by elucidating the regulation process involving the SbbHLH041 TF. In other words, this study exemplifies how inventive thinking can enable the re-customization of [66] pleiotropic plant genetic pathways for the abiotic stress tolerance response, shaped and refined through years of evolution and local adaptation [17], for sustainable environmental solutions in the modern world [67].

## 3. Perspectives

Global food production, plant conservation, and restoration are being jeopardized by abiotic pressures that proliferate at an unprecedented pace, both spatially and temporally [68]. Intricate challenges demand innovative solutions, and as such, modern analytical approaches must be recruited to boost limited or inexistent adaptive donors [69]. If a roadmap is to be drawn for this purpose, the first natural step would be to (i) unify germplasm ecological genetics [70] and in silico genomic mining to target inspirational functionalities in previously characterized gene families [47], as demonstrated by Huang et al. [31]. Yet, when these standing resources prove limited, mainly due to the polygenic architectures that often sustain abiotic stress tolerances, (ii) conventional gene mapping is capable of implementing unforeseen targets for further (iii) validation, and eventually (iv) selection or fast-track edition via gene editing and transgenesis [71]. Interestingly, modern machine learning (ML) resources [72,73] provide transversal perspectives for more efficient mining [74], mapping [75], and validation [76] (step 1–3) of pre-existent and novel candidate gene variants [77]. Additionally, ground-breaking gene editing [78] has emerged as a promising alternative to enhance the speed [79,80] and precision of candidate gene manipulations [81], while evading transgenic boundaries [82], even in a polygenic setup [83]. Exciting times lie ahead at the intersection of these pioneering technologies (i.e., from the fields of molecular biology [84], crop improvement [85,86], conservation [87], and seed protection [88]), an emergent niche that promises to boost plant diversity for abiotic stress tolerance in the face of food insecurity [89], biodiversity loss [68] and the planetary climatic crisis [90].

## 4. Conclusions

In order to tackle the growing abiotic challenges exacerbated by climate change, initiatives from the plant breeding, conservation, and restoration arenas are in critical need of extended adaptation sources, which have been depleted after years of intensified selection and demographic bottlenecks. In this Special Issue, we have assertively gathered cutting-edge research on the discovery, fine tuning, and effective utilization of plant genetic diversity to enhance abiotic stress tolerance features across a wide spectrum of stresses, trait architectures, and species. This accomplishment was made possible by merging the most recent technological advancements in molecular biology, genomics, bioinformatics, and biotechnology. Judging by the featured papers, the path ahead is both inclusive and exciting. Only time will tell whether molecular-based technologies will be capable of effectively leveraging plant diversity at the scale needed to withstand rising abiotic stresses.

## Figures and Tables

**Table 1:** Thematic collection of seven studies in the current Special Issue.

Species	Goal	Sampling	Genotyping	Key Finding	Reference
**Molecular In Silico Characterization**					
*Apocynum* *venetum*	° To characterize in silico AvNAC proteins for abiotic stress responses	Genome sequence of *A. venetum*	WGS and 74 NAC TFs classified in 15 subgroups	*AvNAC58* and *69* are differentially expressed in drought and salt stresses	Huang et al. [31]
**Genetic Mapping**					
Cowpea (*Vigna unguiculata*)	^+^ To unveil the genomic architecture of salt tolerance in cowpea	331 cowpea accessions from USDA-PGRCU tested in greenhouse trials	WGR (14,465,516 SNPs) imputed in GWAS (BLINK)	Identified QTLs and SNPs enable cowpea breeding through MAS	Ravelombola et al. [32]
Sugarcane (*Saccharum officinarum*)	^+^ To detect candidate genes for drought tolerance and agronomic traits	159 diverse sugarcane accessions enriched for Thai lines and varieties	Target enrichment sequencing of 649 gene candidates to drought tolerance	19 pleiotropic genes were related to the drought-tolerance response	Wirojsirasak et al. [33]
**Gene Functional Validation via Expression Analysis**					
Alfalfa (*Medicago sativa*)	^‡^ To identify *Msr* genes and validate their response to abiotic stress	Alfalfa “Xinjiang DaYe” genome sequence and “Zhongmu No. 1”	15 *Msr* genes validated with qRT-PCR	*MsMsr* genes play a role in during salt, drought, and ABA stresses	Zhao et al. [34]
*Phoebe bournei*	^‡^ To identify *LBD* genes and validate their response to abiotic stress	Genome sequence of *P. bournei*, and a mature tree for qRT-PCR	38 putative LBD gene sequences, 5 selected for qRT-PCR	*PbLBDs* were pleiotropic for cold, heat, drought, and salt stress responses	Ma et al. [35]
Sorghum (*Sorghum bicolor*)	^‡^ To map and validate the molecular responses during salt stress	Sorghum cultivar “Lvjuren”	Transcriptome profiling and qRT-PCR	*HKT1;5, CLCc* and *NPF7.3-1* are candidate genes in the salt stress response	Guo et al. [36]
**Trans-Genesis for Gene Functional Validation**					
Sorghum (*Sorghum bicolor*)	^§^ To overexpress *SbEXPA11* to validate its functionality for Cd exposure	Transgenic and WT sorghum cultivar ‘TX430’	qRT-PCR of *SbEXPA11*	*SbEXPA11* is a target to develop phytoremediary sorghum varieties	Wang et al. [37]

The table is sorted top-down by scope and species. The scope is classified as follows: ° molecular in silico characterization, + genetic mapping, ‡ gene functional validation with expression analysis, and § trans-genesis for gene functional validation. Target abiotic stresses are in bold. Abbreviations are enlisted in the following lines. ABA: abscisic acid; BLINK: Bayesian information and linkage disequilibrium iteratively nested keyway [38]; Cd: cadmium; EXPA: α-expansin; CLC: chloride channel; GWAS: genome-wide association study; HKT: high-affinity potassium transporters; LBD: lateral organ boundary domain; MAS: marker-assisted selection; Msr: methionine sulfoxide reductase; NPF: asparagine–proline–phenylalanine (NPF) motif; qRT-PCR: real-time quantitative PCR; QTL: quantitative trait loci; SNP: single-nucleotide polymorphism; TF: transcription factors; USDA-PGRCU: United States Department of Agriculture—Plant Genetic Resources Conservation Unit; WGR: whole-genome re-sequencing; WGS: whole-genome sequencing; WT: wild type.

## Data Availability

For data availability, please see the original articles [31,32,33,34,35,36,37] within the Special Issue “Abiotic Stress Tolerance and Genetic Diversity in Plants” (https://www.mdpi.com/journal/ijms/special_issues/80P9M9NK87 accessed on 24 March 2023).
